# Effects of Straw Particle Size and Physical Forms of Corn in Starter Diets on Growth Performance and Rumen Parameters in Holstein Calves During the Pre-Weaning Period

**DOI:** 10.3390/ani16040643

**Published:** 2026-02-17

**Authors:** Çağdaş Kara, Samet Çevik, Abdülkadir Orman, Nurcan Karslıoğlu Kara, Anna Catharina Berge

**Affiliations:** 1 Department of Animal Nutrition and Nutritional Diseases, Faculty of Veterinary Medicine, Bursa Uludağ University, 16059 Görükle, Bursa, Türkiye; samet.cevik@gmail.com; 2 Department of Zootechnics, Faculty of Veterinary Medicine, Bursa Uludağ University, 16059 Görükle, Bursa, Türkiye; orman@uludag.edu.tr; 3 Department of Animal Science, Faculty of Agriculture, Bursa Uludağ University, 16059 Görükle, Bursa, Türkiye; nkara@uludag.edu.tr; 4 Veterinary Epidemiology Unit, Department of Internal Medicine, Reproduction and Population Medicine, Faculty of Veterinary Medicine, Ghent University, Salisburylaan 133, 9820 Merelbeke, Belgium; anna.berge@ugent.be

**Keywords:** straw particle size, corn processing method, growth performance, rumen fermentation, dairy calves

## Abstract

This study evaluated the effects of straw particle size (short or long) and corn physical form (ground or whole) in diets on growth performance, rumen fermentation and fecal score in calves. Sixty female newborn calves were randomly assigned to one of the four treatments: 90% pelleted starter and 10% short straw (PSS); 70% pelleted starter, 20% whole corn and 10% short straw (PWCSS); 90% pelleted starter and 10% long straw (PLS); 70% pelleted starter, 20% whole corn and 10% long straw (PWCLS). In PSS and PLS treatments, all of the corn was within the pelleted starter. Calves were weaned at 68 days of age. Body weight (BW), wither height and heart girth were measured at 3 and 68 days of age. Feed intakes and fecal scores were measured daily. Rumen fluid and blood samples were collected for rumen pH, rumen volatile fatty acid (VFA) and blood β-hydroxy butyrate (BHB) measurements at 68 days of age. Weaning BW, average daily weight gain (ADG) and weaning wither height were significantly lower in PLS compared to other treatments. Weaning heart girth was significantly lower in PSS and PLS than PWCSS and PWCLS. Feed intake was significantly higher for PWCSS than PWCLS. PWCLS had a significantly lower feed efficiency (starter feed intake/ADG) than PLS. No significant differences were observed for ruminal pH, ruminal acetate and blood BHB among the treatments. In the diets including short straw, ruminal propionate, butyrate and total VFA concentrations were significantly higher for PWCSS than PSS. In the diets including long straw, the ruminal propionate concentration was significantly greater for PLS than PWCLS, and ruminal butyrate and total VFA concentrations were not different for PLS and PWCLS. This study indicated that the effect of corn physical form (ground or whole) on ruminal propionate, butyrate and total VFA concentrations could vary depending on straw particle size. Fecal score was significantly lower in PSS compared to other treatments. In conclusion, long straw combined with pelleted concentrate reduced growth performance in pre-weaning calves. Whole corn inclusion in the diets with long straw increased ADG and weaning BW and improved feed efficiency.

## Simple Summary

This study evaluated the interactive effects of straw particle size (short or long) with two physical forms of corn (ground or whole) in the diets offered as total mixed rations on growth performance of calves during the pre-weaning period. In the diets including short chopped straw, weaning body weight, daily weight gain, starter intake and feed efficiency were similar for the calves fed ground or whole corn. In the diets including long chopped straw, the inclusion of whole corn into the diet led to higher weaning body weight and daily weight gain and better feed efficiency compared with the diet including ground corn. The lowest weaning body weight and daily weight gain were observed in calves fed the total mixed ration with long chopped straw and pelleted concentrate including ground corn. Thus, this study indicated that long chopped straw and pelleted concentrate provision together might not be an appropriate combination to improve growth performance in pre-weaning calves.

## 1. Introduction

During the pre-weaning period, dairy calves face tremendous physiological and metabolic challenges owing to the transition from monogastric to functional ruminal digestion. The transition from milk-fed pre-ruminants to functional ruminants is fundamental for their growth and health performance, and a successful calf rearing program aims to maximize the growth potential of dairy calves early in life [1,2]. Appropriate nutrition management for calves is essential in successful rearing programs for dairy farmers [2,3]. During the pre-weaning period of dairy calves, the physical and metabolic development of the rumen and the subsequent transition from monogastric form to functional ruminal digestion are mainly influenced by the solid feed intake and solid feed composition. Solid feeds for pre-weaning ruminants include starter concentrate feeds in various contents and forms and/or different forage sources. Starter concentrate feeds, especially cereal grains as a starch source, stimulate the rumen microbial population and volatile fatty acid production, which initiates rumen development. Within the scope of rumen development, rumen papillae development is stimulated by the fermentation of starter concentrate producing volatile fatty acids [1,3]. On the other hand, the inclusion of forage into starter feeds during the pre-weaning period triggers early rumination and muscular development of the rumen [3,4,5].

Supplying forage to dairy calves has been a common practice [6,7,8]. Provision of forage might be beneficial in terms of stimulating rumination [4,9], preventing decreases in ruminal pH [5,10], decreasing ruminal parakeratosis [3,5] and the subsequent enhancement of growth performance [5,11,12] in young calves. The effects of forage addition to starter feeds on growth performance and health status in young calves have been variable across studies owing to the source of forage [11,13], the proportion of forage in solid feed [14,15,16], the forage provision method (free-choice provision vs. total mixed ration) and physical characteristics of the starter feed [16,17]. A limited amount of forage in starter the rations of young calves has appeared to positively alter the rumen environment, resulting in increased intake and improved feed efficiency [8,18]. The particle size of forage fed to calves has been investigated. Despite many studies investigating the effects of different particle sizes of forage on rumen and growth performance parameters in young calves, the optimum particle size of forage offered as a free-choice provision or total mixed ration (TMR) has not been clearly determined [6,8,19].

Different types [13,20,21] and/or processing methods [22,23,24] of cereal grains used in starter concentrate feeds affect the physical and metabolic growth of the rumen, the palatability and intake of solid feed and growth performance in young calves. Corn has been extensively incorporated in different forms in calf starter rations: whole [22,25], ground [24,26] and steam-flaked [24,27]. There is a growing interest in using whole grains, especially corn, as a potential alternative strategy instead of forage feeding in calves. Whole grains can lead to increased particle sizes in the calf starter diet and probably results in better rumen health and function by preventing decreased ruminal pH, causing papillae keratinization [25,28]. Lesmeister and Heinrichs [22] observed that calves fed starter feed with whole corn had higher rumen pH than calves offered starter feeds with dry-rolled, roasted-rolled or steam-flaked corn. Kamyab-Fard et al. [25] reported that ruminal pH was affected by different levels of whole corn in starter feed, and increasing the amount of whole corn replaced with ground corn led to increased ruminal pH in young calves fed forage-free starter feeds. A previous study by Gholizadeh et al. [26] investigated the effects of combining corn grain forms (ground vs. whole) with forage (0 vs. 10%) in calf starters. They reported that ruminal pH, VFA concentrations, ADG and feed efficiency were not affected by the physical form of corn (ground vs. whole) in the starter diets, regardless of forage provision, during the pre-weaning period. In that study [26], the combination of whole corn and forage led to a lower starter feed intake without influencing performance during the pre-weaning period. These results suggest that dairy farmers can use whole corn grain in starter feeds without any negative growth or health effect, which is important, since grinding corn is an additional step and expenditure [26].

Maximizing starter feed intake and rumen growth have been positively correlated with weight gain in pre-weaned calves, which is partly related to a greater milk production during the first lactation [29,30]. Since a great variation has been found among the calf studies examining the effects of different forms of starter feeds (ground, pelleted, and textured) and/or different forage provision methods (forage-free starter, free-choice provision or TMR), there is insufficient evidence for a recommended starter diet to improve the growth rates of dairy calves [13]. Therefore, our study aimed to investigate the interactive effects of different straw particle sizes with two different physical forms of corn grain (whole or ground) in the starter diets on growth performance and rumen fermentation of Holstein calves during the pre-weaning period. We tested the hypothesis of whether calves fed different physical forms of corn responded differently depending on particle size of straw in the starter diets.

## 2. Materials and Methods

This study was carried out at a commercial dairy farm (Tarfaş Holstein Dairy Farm) in Bursa, Türkiye. This study was conducted under a protocol approved by the Animal Care and Use Committee of Bursa Uludağ University (decision no. 2022-03/13).

### 2.1. Calves, Management and Experimental Diets

Sixty Holstein female dairy calves (36.5 ± 3.5 kg of body weight at 3 days of age) were randomly assigned to one of the four treatments (*n* = 15 calves per treatment) in a completely randomized design, with a 2 × 2 factorial arrangement of treatments, including (1) starter diet containing 90% pelleted calf starter (P; 34.60% ground corn) and 10% straw with a short particle size on an as-fed basis (PSS); (2) starter diet containing 70% pelleted calf starter (15.18% ground corn) plus 20% whole corn (PWC) and 10% straw with a short particle size on an as-fed basis (PWCSS); (3) starter diet containing 90% pelleted calf starter (P; 34.60% ground corn) and 10% straw with a long particle size on an as-fed basis (PLS); (4) starter diet containing 70% pelleted calf starter (15.18% ground corn) plus 20% whole corn (PWC) and 10% straw with a long particle size on an as-fed basis (PWCLS). In the PSS and PLS treatments, all amounts of corn grain were within the pelleted calf starter.

Immediately after birth, calves were separated from their dams and housed in individual pens (1.2 × 2.0 m) bedded with clean straw, which was refreshed daily. The front of each pen had 2 openings for access to pails mounted on the outside. Water was provided ad libitum in one pail and solid feed was provided ad libitum in the second pail. The calves were fed 5 L of previously frozen or fresh colostrum (Brix > 22%) during the first 12 h of life (2.5 L by 1 h after birth and 2.5 L at 12 h after the first feeding). On day 2 of life, calves received transition milk (5 L/day) in three equal meals. According to the farm’s protocol, all calves were fed 6 L/day of pasteurized whole milk in plastic feeding buckets from 3 to 14 days of age three times daily; 7.5 L/day from 15 to 42 days of age three times daily; 6 L/day from 43 to 49 days of age three times daily; 5 L/day from 50 to 56 days of age twice daily; 4 L/day from 57 to 63 days of age twice daily; and 2 L/day from 64 to 68 (the end of the study) days of age once daily. Milk was sampled weekly and analyzed for fat, crude protein, lactose and total solids using an infrared analyzer (FOSS, MilkoScan^TM^ FT3, Hillerød, Denmark). The average composition of offered milk was 3.65 ± 0.06% fat, 3.29 ± 0.07% crude protein, 4.60 ± 0.07% lactose and 12.51 ± 0.13% total solids. The calves used in our study were in good health condition prior to beginning the experiment (3 days of age). All calves were weaned at 68 days of age (the end of the study).

The calves had free access to starter diet and water throughout the study (from 3 to 68 days of age). The starter diets provided as TMR were offered once daily at 08:00 h to result in at least 5% residual feed (orts) over a 24 h period. Before the experiment, wheat straw was chopped to obtain short or long particle sizes (short straw or long straw) using a TMR mixer (Ak Agricultural Machinery Industry Co., İzmir, Türkiye). The particle size distribution of wheat straw with a short particle size (short straw) and long particle size (long straw) are presented in Table 1. Pelleted calf starters used in TMRs with or without whole corn were formulated to have the same ingredients. Two different pelleted starters were prepared with respect to the formulation and chemical compositions so that experimental starter diets as a TMR had similar nutrient contents. The main ingredients (cereal grains, protein sources and by-products) in the pelleted starters were ground to pass through a 2 mm mesh. The pelleted calf starters were 20 mm in length and 3 mm in diameter. The ingredient and nutrient compositions of the pelleted starters used in TMRs with or without whole corn are given in Table 2. The ingredient and chemical compositions of experimental starter diets (PSS, PLS, PWCSS, and PWCLS) are presented in Table 3. Experimental diets were formulated to be iso-energetic and iso-nitrogenous using NRC (2001) software. The starter diets offered as a TMR were prepared daily in a big bucket by the manual mixing of diet components. Experimental starter diets were prepared from the same batches of wheat straw and corn grain to prevent the effects of nutritional variation in straw and corn variety on the results. The calves were individually monitored twice daily by a farm veterinarian. Sick calves were treated by a veterinarian according to regular farm protocols.

### 2.2. Measurements, Sample Collection and Analyses

The particle size distribution of chopped wheat straw used in 4 experimental diets was determined using the Penn State Particle Separator (PSPS, Nasco, Fort Atkinson, WI, USA), equipped with 3 sieves (19, 8, and 1.18 mm) and a bottom pan. Chopped wheat straw samples (200 g) were collected 4 times throughout the study from each particle size group (short and long) for particle size distribution analysis. The pelleted starters used in TMRs with or without whole corn and experimental starter diets (PSS, PLS, PWCSS, and PWCLS) were ground using a laboratory mill through a 1 mm screen for chemical analyses and then dried in an oven at 105 °C overnight. Nutrient analyses (crude protein, ether extract, starch and crude ash) of the pelleted starter and experimental starter diet samples were performed according to AOAC [32], and neutral detergent fiber (NDF), acid detergent fiber (ADF) and acid detergent lignin (ADL) analyses of the pelleted starters and experimental TMRs were performed as described by Van Soest et al. [33] in the Department of Animal Nutrition and Nutritional Diseases, Bursa Uludağ University Veterinary Faculty, Bursa, Türkiye.

Body weight (BW) and structural growth measurements (wither height, distance from the base of the front feet to withers and heart girth, and circumference of the chest) were measured at 3 days of age (the beginning of the study) and 68 days of age (the end of the study) prior to the morning feeding. Average daily weight gain (ADG) was calculated as the difference between two BW measurements divided by days. Starter feed (TMR) intake was measured daily on an individual basis throughout the study by subtracting refusals from the amounts offered. Feed efficiency (FE) was calculated by dividing average daily starter intake (kg) by ADG (kg).

Rumen fluid samples from each calf were obtained using a stomach tube fitted to a vacuum pump at 68 days of age (4 h after feeding). The first 15 mL of rumen fluid was discarded to eliminate the potential saliva contamination and then the rest of rumen fluid was collected. Thereafter, the samples were squeezed through 4 layers of cheesecloth, and then rumen fluid pH was immediately measured using a portable pH meter (Inolab pH, serial no: 00200018, pH Electrode SenTix 41, D-82362, Weilheim, Germany). For the analysis of volatile fatty acids (VFAs), 4 mL of rumen fluid samples were acidified with 1 mL of 25% meta-phosphoric acid in plastic tubes. After the samples were centrifuged at 5000× *g* for 10 min, the supernatants were stored at −20 °C until the analysis. Ruminal VFA concentrations were determined using gas chromatography (Hewlett Packard Agilent Technologies 6890N Network GC System, Serial CN10447002, Beijing, China). A column (6 × 2 mm ID glass) was packed with 10% SP-1200/1% H_3_PO_4_ 80/100 Chromosorb WAW (Supelco, Bellefonte, PA, USA). Carrier gas (N_2_) flow was 30 mL/min, inlet temperature was 175 °C, detector temperature was 170 °C, and column temperature was 110 °C. Detection was carried out by flame ionization.

Blood samples were taken from the jugular vein of all calves into 10 mL evacuated tubes 3 h after morning feeding at 68 days of age. The concentrations of blood β-hydroxy butyrate (BHB) were immediately measured using blood ketone test strips (Abbott Diabetes Care Ltd., Range Road Witney, Oxfordshire, UK) with the Abbott Precision Xtra^TM^ meter (Abbott Diabetes Care Ltd., Range Road Witney, Oxfordshire, UK).

Before morning feeding, fecal samples were collected daily from each calf by retrieval from the rectum. Fecal samples were scored regarding consistency by the same researcher according to the following system: 1 = normal, 2 = soft to loose, 3 = loose to watery, 4 = watery, mucous, slightly bloody, and 5 = watery, mucous and bloody, as described by Heinrichs et al. [34].

### 2.3. Statistical Analysis

Data of body weight, ADG and average daily starter feed intake (from 3 to 68 days of age), feed efficiency, structural growth measurements (wither height, hearth girth and the changes in wither height and hearth girth), ruminal pH, ruminal VFA concentrations (acetate, propionate, butyrate and total), blood β-hydroxy butyrate concentration and average daily fecal score (from 3 to 68 days of age) were analyzed by General Linear Models, where the groups (main groups; straw with short particle and straw with long particle and subgroups; pelleted calf starter and pelleted calf starter plus whole corn) were included in the models as fixed factors. The normality of the data distribution was analyzed using the Kolmogorov–Smirnov test. A full factorial model was used as the model for interaction effects between the straw particle size and physical forms of corn, and the Tukey test was used as a post-test for comparing means. The threshold of significance was set at *p* ≤ 0.05. Trends were declared when 0.05 < *p* ≤ 0.10. All statistical analyses were conducted by using SPSS 29.0 software (IBM, Chicago, IL, USA).

## 3. Results

All calves used in our study consumed all of the milk offered daily during the suckling period. Excluding diarrhea, no health problems such as respiratory diseases were observed and no mortality occurred during the study. The number of calves treated for diarrhea was one for the PSS, PWCSS and PLS treatments and two for the PWCLS treatment.

**Figure 1:**
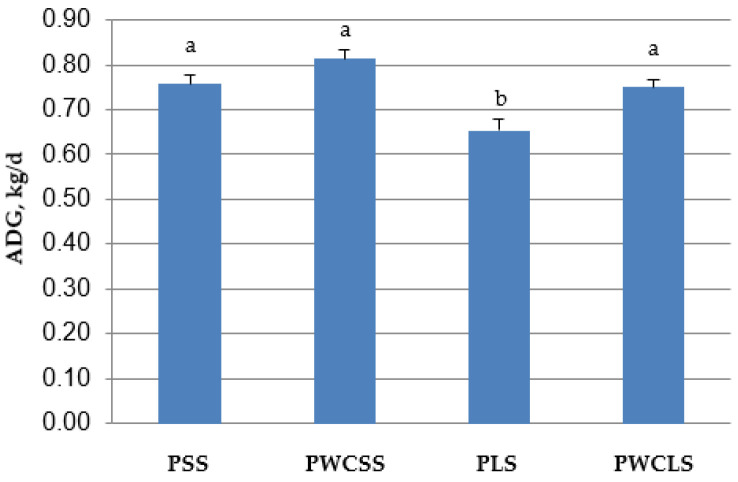
Mean average daily weight gain (ADG) from 3 days to 68 days of age in PSS, PWCSS, PLS and PWCLS treatments. Each bar represents the means and standard errors (*n* = 15). Abbreviations: PSS, 90% pelleted calf starter and 10% straw with a short particle size; PWCSS, 70% pelleted calf starter plus 20% whole corn and 10% straw with a short particle size; PLS, 90% pelleted calf starter and 10% straw with a long particle size; PWCLS, 70% pelleted calf starter plus 20% whole corn and 10% straw with a long particle size. Different letters above the bars show a significant difference among the treatments (*p* ≤ 0.05).

**Figure 2:**
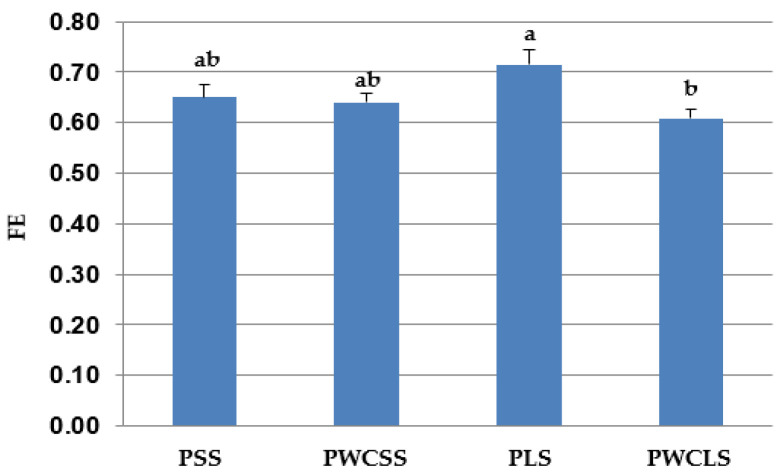
Mean feed efficiency (FE = kg of mean starter feed intake/kg of mean average daily weight gain) in PSS, PWCSS, PLS and PWCLS treatments. Each bar represents the means and standard errors (*n* = 15). Abbreviations: PSS, 90% pelleted calf starter and 10% straw with a short particle size; PWCSS, 70% pelleted calf starter plus 20% whole corn and 10% straw with a short particle size; PLS, 90% pelleted calf starter and 10% straw with a long particle size; PWCLS, 70% pelleted calf starter plus 20% whole corn and 10% straw with a long particle size. Different letters above the bars show a significant difference among the treatments (*p* ≤ 0.05).

The results on BW, ADG, TMR intake and feed efficiency are presented in Table 4 (Figure 1 for ADG and Figure 2 for feed efficiency). BW at 3 days of age (the beginning of the study) was similar for all treatments (*p* > 0.05). BW at 68 days of age (weaning time) and ADG were lower for the calves fed the PLS treatment than those fed the other treatments (*p* < 0.05). Weaning BW and ADG were affected by the particle size of straw, physical form of corn (ground vs. whole) and their interaction (*p* < 0.01). Starter feed intake was higher for the PWCSS treatment than the PWCLS treatment (*p* < 0.05). The interaction between the straw particle size and corn physical form was significant (*p* = 0.05) for the starter feed intake. PWCSS had the highest starter feed intake among the treatments. There was a difference in feed efficiency (kg of mean starter feed intake/kg of mean ADG) between PLS and PWCLS treatments. PWCLS feeding resulted in lower feed efficiency than PLS feeding (*p* < 0.05). An interaction was observed between the straw particle size and corn physical form in starter diets for feed efficiency (*p* = 0.01).

Structural growth measurements (wither height and heart girth) data are shown in Table 5. Wither height and heart girth measurements at 3 days of age were similar for all treatments (*p* > 0.05). Wither height at 68 days of age and the change in wither height were lower (*p* < 0.05) for the PLS treatment than the other treatments (PSS, PWCSS and PWCLS). Wither height (68 days of age) and the change in wither height were affected by the interaction between the straw particle size and corn physical form (*p* < 0.01). Heart girth (68 days of age) and the change in heart girth were lower for the PSS and PLS treatments than the PWCSS and PWCLS treatments (*p* < 0.05). There was a tendency for the effect of the straw particle size on heart girth at 68 days of age (*p* = 0.08) and heart girth change (*p* = 0.07) in favor of the calves fed the diets with short straw. Heart girth (68 days of age) and the change in heart girth were influenced by the physical form of the corn in the starter diets (*p* < 0.01) in favor of the calves fed the diets containing whole corn. The interaction between the straw particle size and physical form of corn in starter diets was significant (*p* < 0.01) for heart girth (68 days of age) and the change in heart girth.

**Figure 3:**
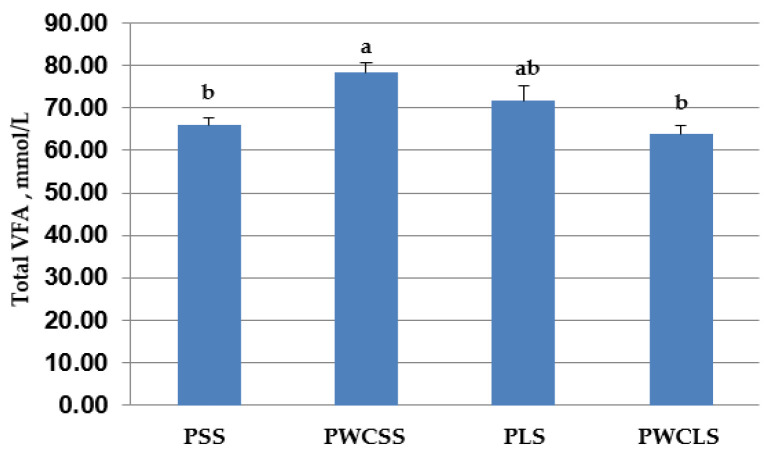
Mean total VFA (acetate + propionate + butyrate) concentrations in PSS, PWCSS, PLS and PWCLS treatments. Each bar represents the means and standard errors (*n* = 15). Abbreviations: PSS, 90% pelleted calf starter and 10% straw with a short particle size; PWCSS, 70% pelleted calf starter plus 20% whole corn and 10% straw with a short particle size; PLS, 90% pelleted calf starter and 10% straw with a long particle size; PWCLS, 70% pelleted calf starter plus 20% whole corn and 10% straw with a long particle size. Different letters above the bars show a significant difference among the treatments (*p* ≤ 0.05).

Ruminal pH and VFA concentrations at 68 days of age are presented in Table 6 (and Figure 3 for total VFA). Ruminal pH value and ruminal acetate concentration did not differ among the treatments (*p* > 0.05). In the diets including short straw, ruminal propionate, butyrate and total VFA concentrations were higher for the PWCSS treatment than the PSS treatment (*p* < 0.05). In the diets including long straw, the ruminal propionate concentration was higher for the PLS treatment than the PWCLS treatment (*p* < 0.05), while ruminal butyrate and total VFA concentrations were similar for the PLS and PWCLS treatments (*p* > 0.05). Ruminal propionate concentration was affected by the interaction between the straw particle size and corn physical form (*p* < 0.01). Ruminal butyrate concentration was greater (*p* < 0.05) for the PWCSS treatment than the other treatments (PSS, PLS and PWCLS). Ruminal total VFA concentration was higher (*p* < 0.05) for the PWCSS treatment than the PSS and PWCLS treatments. The interaction between the straw particle size and physical form of corn in starter diets was significant (*p* < 0.01) for ruminal butyrate and total VFA concentrations.

**Figure 4:**
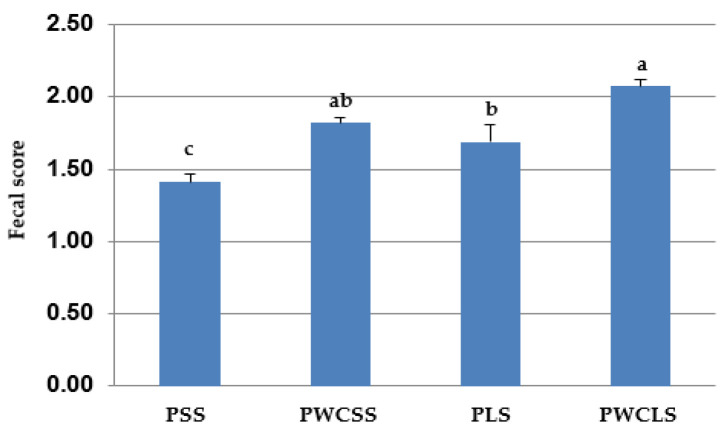
Mean fecal score in PSS, PWCSS, PLS and PWCLS treatments. Each bar represents the means and standard errors (*n* = 15). Abbreviations: PSS, 90% pelleted calf starter and 10% straw with a short particle size; PWCSS, 70% pelleted calf starter plus 20% whole corn and 10% straw with a short particle size; PLS, 90% pelleted calf starter and 10% straw with a long particle size; PWCLS, 70% pelleted calf starter plus 20% whole corn and 10% straw with a long particle size. Different letters above the bars show a significant difference among the treatments (*p* ≤ 0.05).

Blood BHB concentration at 68 days of age and fecal score results are shown in Table 7 (and Figure 4 for the fecal score). Blood BHB concentration did not differ among the treatments (*p* > 0.05). Blood BHB concentration was not influenced by the straw particle size (*p* = 0.66), corn processing method (*p* = 0.18) and their interaction (*p* = 0.41). Mean fecal score from 3 to 68 days of age was lower (*p* < 0.05) in the PSS treatment than the other treatments (PSS, PLS and PWCLS). An interaction was observed between the straw particle size and physical forms of corn in starter diets for mean fecal score (*p* < 0.01).

## 4. Discussion

### 4.1. Growth Performance

According to the results of our study, the PLS treatment had the lowest weaning BW and ADG compared with the other treatments (PSS, PWCSS and PWCLS). This finding could be partly explained by the lower starter feed intake for the PLS treatment than the PSS and PWCSS treatments, as the starter feed intake has been positively correlated with ADG in pre-weaning calves [6,22,26]. However, although the PLS and PWCLS treatments had nearly identical feed intake, weaning BW and ADG remained lower in calves receiving the PLS treatment compared with the PWCLS treatment. This was likely attributable to reduced nutrient digestibility in the PLS treatment relative to the PWCLS treatment.

In dairy calf feeding, supplementation of forage to the starter feed has been a common practice. However, data are limited about how different particle sizes of forage affect calf performance in free-choice provision or the TMR feeding regimen [8,35,36]. In the current study, weaning BW, ADG and starter feed intake were affected by the particle size of straw, regardless of physical forms of corn in starter diets. Omidi-Mirzaei et al. [6] investigated the interactive effects of the forage source and forage particle size as a free-choice provision on the growth performance of calves fed texturized starters. In contrast to our results, Omidi-Mirzaei et al. [6] reported that the particle size of forage did not affect weaning BW, ADG and total feed intake (forage intake and starter intake) during the pre-weaning period, which could be due to the differences in the forage provision method and the particle sizes of utilized forage between ours and that study [6]. Also, Bagheri et al. [8] observed that weaning BW, ADG, starter intake and forage intake were not influenced by the straw particle size as a free-choice provision to calves during the pre-weaning period. Similar to the previous studies mentioned above [6,8], feed efficiency was not affected by the straw particle size in our study, regardless of physical forms of corn in starter diets. Montoro et al. [37] investigated the effects of 10% coarsely chopped grass hay or 10% finely ground grass hay in TMR containing 90% crumb starter concentrate on the performance and apparent digestibility of young calves from 2 to 56 days of age. In that study [37], there was a tendency for improved feed efficiency in the calves fed chopped hay compared with those fed ground hay. Montoro et al. [37] reported that the total tract apparent digestibility of dry matter, crude protein, NDF and ADF during the week after weaning were greater in calves offered the diet including chopped hay than in those fed the diet with ground hay. This result could explain the tendency for an improved feed efficiency in calves fed TMR with chopped hay. According to Montoro et al. [37], the particle size of forage might play an important role in nutrient digestibility. Our result regarding feed efficiency was contrary to that of Montoro et al. [37] when we evaluated the effects of the particle size of straw in the starter diets, regardless of corn physical form. These conflicting results were probably due to the differences in the type and particle sizes of utilized forages and forms (crumb starter vs. pelleted or textured starter) of utilized starter concentrates between ours and that study [37].

It has been demonstrated that the physical form of the starter feeds—pelleted, texturized or ground [5,13,38]—and processing methods of cereal grains used in starter diets [13,22,24] can affect the starter intake and/or growth performance in young calves. In our study, weaning BW and ADG were affected by the physical form of corn despite there being no effect of ground versus whole corn on the starter feed intake, regardless of the forage particle size in TMR. This result could explain the effect of the physical form of corn on feed efficiency. Gholizadeh et al. [26] investigated the interactive effects of forage provision (0 or 10% of chopped straw) and the physical form of corn (ground or whole) in the starter diets (mashed starter compared to a texturized starter containing whole grain) on growth performance of young calves. Contrary to our study, Gholizadeh et al. [26] reported that weaning BW, ADG and feed efficiency during the pre-weaning period were not influenced by physical form of corn in the starter diets, regardless of forage provision. In addition, Gholizadeh et al. [26] found that the starter feed intake was higher for the calves offered the diet with ground corn than those offered the diet with whole corn during the pre-weaning period, when the calves were fed TMR containing 10% of chopped wheat straw. These conflicting results between ours and that study [26] were probably due to the differences in the particle sizes of straw used in starter diets because different particle sizes of forage could affect the growth performance and/or starter feed intake of young calves [13,18,19]. The particle sizes of forage in our TMRs with both short and long particle sizes of straw were longer than those in the study conducted by Gholizadeh et al. [26]. In contrast to our results, Kamyab-Fard et al. [25] indicated that ADG and feed efficiency were similar for the calves fed the diets with or without whole corn when comparing forage-free starter diets containing 60% ground corn versus 40.2% ground corn and 19.8% whole corn. This discrepancy between ours and that study [25] was probably due to the differences in the forage provision and/or form and composition of starter diets, such as amount of corn and the particle size distribution.

In our study, weaning wither height and the change in wither height were lower for the calves fed PLS than those fed PSS, PWCSS or PWCLS, and were similar for PSS, PWCSS and PWCLS. Our results related to wither height were congruent with the findings of ADG and weaning BW. Rincker et al. [39] found that wither height at weaning was positively correlated with weaning BW and ADG during the pre-weaning period in Holstein female calves. In the diets including short straw, weaning heart girth and heart girth changes were greater for the PWCSS treatment (with whole corn) than the PSS treatment. Similarly, in the diets including long straw, weaning heart girth and heart girth changes were higher in the PWCLS treatment (with whole corn) than the PLS treatment. Similar results were observed for weaning BW. Silva et al. [40] indicated that body weight could be reliably estimated using heart girth measurements in pre-weaned Holstein–Friesian calves aged 1 to 90 days, which showed the relationship between body weight and heart girth.

### 4.2. Ruminal pH and Volatile Fatty Acids

In the current study, the mean ruminal pH at 68 days of age ranged from 5.76 to 6.06 in neonatal calves and did not differ among the treatments. Supplementation of forage to the starter feeds prevents decreases in ruminal pH and ruminal acidosis in young calves [5,8,10,16]. Similar to studies in mature ruminants, changes in the particle size of forage have been mainly investigated as one of the most known physical characteristics of forage to affect the ruminal pH of young calves. Nemati et al. [14] observed that ruminal pH was affected by the particle size of alfalfa hay mixed with finely ground concentrates, regardless of the level of forage (12.5 or 25%) in TMR, and ruminal pH was higher for the calves offered a medium particle size of forage than those offered a fine particle size of forage. In contrast with the results of Nemati et al. [14], in our study, ruminal pH was not influenced by the particle size of straw, which was probably due to the differences in the level of utilized forages and the form of starter concentrates between ours and that study [14]. Similar to the result of our study, Omidi-Mirzaei et al. [6] reported that the particle size of forage as a free-choice provision did not affect ruminal pH values in dairy calves. In addition, Bagheri et al. [8] also found that ruminal pH at weaning was not influenced by different particle sizes of wheat straw provided as a free-choice provision to dairy calves. Under the conditions of our experimental design, differences in the particle size distribution of wheat straw between treatments had no effects on ruminal pH at weaning, which might be related to the greater chewing activity in calves compared to cows, and consequently a decrease in the particle size of forage entering the rumen. Also, the calves fed the starter diets including different particle sizes of straw might have sorted for similar particle sizes of straw, thereby showing similar ruminal pH values [8].

Whole corn grain can lead to increased particle sizes in the starter diet and probably result in better rumen health by preventing decreases in the ruminal pH of young calves. Therefore, whole corn grain inclusion in calf starter diets can be considered as a potential alternative of forage feeding to modulate ruminal pH and improve rumen health [25,28]. Kamyab-Fard et al. [25] observed that ruminal pH was influenced by different levels of whole corn in starter feed and increasing the amount of whole corn replaced with ground corn led to increased ruminal pH in young calves fed forage-free starter feeds. In our study, the presence of a forage source in experimental starter diets may have caused the lack of effect of whole corn on ruminal pH. Similar to the result of our study, Gholizadeh et al. [26] reported that ruminal pH values were not affected by different physical forms of corn (ground or whole) in the starter diets mixed with 10% chopped wheat straw in Holstein calves. The high level of milk feeding could lead to lower mean solid feed intake during the pre-weaning period [26,41], which may have caused the lack of possible effect of the straw particle size and physical form of corn in starter diets on ruminal pH in our study, where calves were fed 7.5 L/day milk from 15 to 42 days of age. For instance, the particle size of forage did not influence ruminal pH in the calves fed 6 L/day milk from 3 to 44 days of age [6]. In addition, Gholizadeh et al. [26] found that ruminal pH was not affected by wheat straw inclusion or corn grain physical form in the calves fed 8 L/day milk from 11 to 45 days of age and 6 L/day milk from 46 to 55 days of age.

In the present study, ruminal acetate concentrations did not differ among the treatments. Molaei et al. [42] reported that ruminal acetate concentrations were not affected by the physical form (10% medium chopped vs. 10% pelleted) of alfalfa hay mixed with starter concentrate in Holstein calves. In addition, Omidi-Mirzaei et al. [6] observed that the particle size of forage did not affect ruminal acetate concentrations in dairy calves. Gholizadeh et al. [26] reported that the ruminal acetate concentrations of dairy calves were not influenced by different physical forms of corn (ground or whole) in the starter diets containing 10% chopped wheat straw. Additionally, Gholizadeh et al. [26] found that ruminal acetate concentrations were higher for calves fed diets with straw compared to those fed diets without straw when considering forage inclusion (0 or 10% of chopped straw) into the starter concentrate. Forage provision during the pre-weaning period can promote cellulolytic microbial growth and results in increased acetate production in the rumen of calves [43,44]. In our study, similar ruminal acetate concentrations for all treatments could be explained by the same level of wheat straw used in the experimental diets.

In the present study, ruminal propionate, butyrate and total VFA concentrations at weaning were affected by the interaction between the straw particle size and corn physical form. In the diets including short straw, ruminal propionate, butyrate and total VFA concentrations were higher for the treatment with whole corn (PWCSS) than the treatment without whole corn (PSS). On the other hand, in the diets including long straw, the ruminal propionate concentration was greater in the calves fed the diet without whole corn (PLS) compared to the calves fed the diet with whole corn (PWCLS), and ruminal butyrate and total VFA concentrations were similar for the PLS and PWCLS treatments. These results suggest that the effect of the physical form of corn (ground or whole) on ruminal propionate, butyrate and total VFA concentrations could vary depending on the particle size of straw in starter diets. This could be due to the differences in the particle size distribution of starter diets, resulting in feed sorting. In contrast to the results of our study, Gholizadeh et al. [26] reported that ruminal propionate, butyrate and total VFA concentrations of Holstein calves were not influenced by the different physical forms of corn (ground or whole) in the starter diets containing 10% chopped straw with the same particle size. These conflicting results between ours and that study [26] were probably due to the effect of the forage particle size, as mentioned above. In the study by Kamyab-Fard et al. [25], when comparing forage-free starter diets containing 60% ground corn versus 40.2% ground corn and 19.8% whole corn, ruminal butyrate and total VFA concentrations were similar for the calves fed the diets with or without whole corn, while there was a tendency for a lower ruminal propionate concentration in the calves offered the diet including whole corn compared to those fed the diet without whole corn. The results of ruminal propionate, butyrate and total VFA concentrations in that study [25] were similar to our results for the diets including long straw. However, in the diets including short straw, our results for ruminal propionate, butyrate and total VFA concentrations were different from the findings of the study by Kamyab-Fard et al. [25], and the reasons for the different results could not be explained. The different results for whole corn inclusion in calf starter diets on ruminal VFA concentrations might be related to the forage provision (forage-free or offering forage) and/or the absorption rate of VFAs produced in the rumen of young calves, since the absorption of VFA can be a factor affecting its concentration in the rumen fluid [25,45]. In fact, the numerically lower ruminal pH (5.80) for the PWCSS treatment may have negatively influenced the VFA absorption compared with the PSS treatment (6.06). Therefore, higher ruminal propionate, butyrate and total VFA concentrations may have been found in the PWCSS treatment than the PSS treatment in the spot sampling of rumen fluid 4 h after feeding, which was probably due to their slow absorption by the rumen wall [25,46]. Similarly, Kamyab-Fard et al. [25] indicated that the concentrations of ruminal total VFA were increased when ruminal pH values decreased in Holstein calves. In addition, Kitkas et al. [47] reported that the ruminal propionate concentration was elevated when ruminal pH values decreased in dairy cows.

### 4.3. Blood BHB and Fecal Score

In our study, the blood BHB concentration at 68 days of age was not influenced by the particle size of straw, while the straw particle size affected the starter feed intake. This was in contrast to previous studies [48,49,50] that reported blood BHB concentration positively correlated with solid feed intake in calves. There was no effect of the physical form of corn in starter diets on both the blood BHB concentration and starter feed intake, regardless of the forage particle size. Similar to our study, Bagheri et al. [8] reported that the particle size of wheat straw offered as a free-choice provision to calves did not affect the blood BHB concentration during the pre-weaning or post-weaning periods. In contrast to our result, Nemati et al. [14] found that blood BHB concentrations at 70 days of age were affected by the particle size of forage in the diets offered as a TMR and were greater in calves fed medium compared to fine particle sizes of forage, regardless of the level of forage. These different results regarding the BHB concentration might be due to the fact that calves were weaned earlier, thus the greater solid feed intake in the study conducted by Nemati et al. [14] compared with our study. Similar to our study, Gholizadeh et al. [26] found that the physical form of corn in starter diet (ground vs. whole) did not affect the blood BHB concentration during the pre-weaning or post-weaning periods when the calves were fed the TMR containing 10% of chopped wheat straw. In addition, Kamyab-Fard et al. [25] also reported that the serum BHB concentration was similar for the calves fed the diets with or without whole corn when comparing forage-free starter diets containing 60% ground corn versus 40.2% ground corn and 19.8% whole corn.

Blood BHB is a measure of rumen epithelial metabolic activity and indicates the conversion of ruminal butyrate to β-hydroxy butyrate. Butyrate is absorbed by the ruminal epithelium and oxidized into BHB [20,49]. Deelen et al. [49] reported that blood BHB results were positively associated with ruminal butyrate in dairy calves around weaning. According to the results of our study, there was a consistent relationship between blood BHB and ruminal butyrate concentrations for all treatments except for PWCSS. Although the ruminal butyrate concentration at 68 days of age was higher in the PWCSS treatment than in the other treatments (PSS, PLS and PWCLS), the blood BHB concentration at 68 days of age did not differ among the treatments, which might suggest that increased ruminal butyrate concentration for the PWCSS treatment had a limited effect on rumen epithelium maturation when considering the results of the blood BHB concentration as an indicator of rumen epithelium development. Similar results for feed efficiency between the PWCSS treatment and the other treatments might confirm this suggestion, since rumen papillae development is important for nutrient and VFA absorption [43,51].

In the current study, a higher fecal score indicated the formation of softer feces. Mean fecal scores were higher in the calves fed straw with a long particle size than those fed straw with a short particle size. Calves fed the starter diets with whole corn (both PWCSS and PWCLS) had a higher mean fecal score. The highest mean fecal score was observed in the PWCLS treatment. In the present study, the number of calves treated for diarrhea was one for the PSS, PWCSS and PLS treatments and two for the PWCLS treatment. The increase in the mean fecal score for the PWCLS treatment had no clinical importance when considering the number of calves treated for diarrhea. In contrast to our results, Mirzaei et al. [19] observed that the fecal score was not influenced by the particle size of alfalfa hay in dairy calves during the pre-weaning period, when alfalfa hay was mixed with finely ground concentrates and offered as a TMR. This conflicting result between ours and that study [19] might be due to the differences in the type and particle sizes of used forage and/or the differences in the level of forage inclusion and the form of concentrates in starter diets. In our study, the mean fecal score was affected by the physical form of corn in starter diets. Contrary to the findings of our study, Gholizadeh et al. [26] found that the fecal score was not affected by the corn grain physical form (ground or whole) in the starter diets of Holstein calves fed TMR containing 10% of chopped wheat straw during the pre-weaning period. Kamyab-Fard et al. [25] also reported that the fecal score was not associated with different levels of whole corn in forage-free starter feeds. This discrepancy between the result of our study and that of others [25,26] was probably due to the differences in the form and composition of starter diets, such as amount of corn and the particle size distribution.

This study has some limitations due to the relatively short experimental period and the use of specific straw particle sizes and a specific corn type in the experimental diets. Further research with longer durations and/or different straw particle sizes and corn varieties is needed to evaluate and confirm these findings.

## 5. Conclusions

In this study, the starter diet containing long straw and pelleted concentrate (PLS treatment) led to lower weaning BW and ADG compared to other diets (PSS, PWCSS and PWCLS treatments). This suggested that long straw combined with a pelleted concentrate might not be a suitable combination for starter diets offered as a TMR. Whole corn inclusion led to increased weaning BW, increased ADG and improved feed efficiency in the diets including long straw. In the diets containing short straw, the inclusion of whole corn into calf starter (feeding a texturized starter) did not affect weaning BW, ADG and feed efficiency in pre-weaning calves. Starter feed intake was higher for the calves fed the diets including short straw compared to long straw. The physical form of corn in starter diets did not affect the starter feed intake. Ruminal pH, ruminal acetate and blood BHB values at weaning were not influenced by the particle size of straw and the physical form of corn. In the diets with short straw, ruminal propionate, butyrate and total VFA concentrations were higher for the treatment with whole corn (PWCSS) than the treatment without whole corn (PSS). In the diets including long straw, ruminal propionate concentration was greater in the calves fed the diet without whole corn (PLS) compared to the calves fed the diet with whole corn (PWCLS), whereas ruminal butyrate and total VFA concentrations were similar. These results indicated that the effect of the physical form of corn (ground or whole) on ruminal propionate, butyrate and total VFA concentrations could vary depending on the particle size of straw in starter diets. The fecal score was affected by the particle size of straw and the physical form of corn. Calves fed the starter diets with whole corn (PWCSS and PWCLS) had a higher mean fecal score (softer feces). The highest mean fecal score was observed in the PWCLS treatment. The findings of our study will usefully contribute to the data on the particle size of forage and the physical forms of corn in starter diets offered to dairy calves during the pre-weaning period.

## Figures and Tables

**Table 1:** Particle size distribution of chopped wheat straw fed to calves according to The Penn State Particle Separator (PSPS).

Particle Size Distribution, %	Short Straw	Long Straw
>19.0 mm		
Minimum	16.50	51.00
Maximum	17.67	54.70
Mean ± standard deviation	17.16 ± 0.50	53.06 ± 1.58
8.0–19.0 mm		
Minimum	45.12	19.57
Maximum	47.90	24.60
Mean ± standard deviation	46.41 ± 1.24	22.54 ± 2.13
1.18–8.0 mm		
Minimum	24.30	16.00
Maximum	26.47	18.86
Mean ± standard deviation	25.20 ± 0.92	17.26 ± 1.19
<1.18 mm		
Minimum	10.43	6.10
Maximum	12.09	7.83
Mean ± standard deviation	11.23 ± 0.79	7.13 ± 0.74

**Table 2:** Ingredient and nutrient compositions of the pelleted starters used in TMRs with or without whole corn.

Ingredient, % of as-Fed	Pelleted Starter in TMR Without Whole Corn	Pelleted Starter in TMR with Whole Corn
Corn	34.60	15.18
Barley	5.00	6.91
Soybean meal	16.77	20.96
DDGS ^1^	11.40	14.85
Sunflower meal	3.86	4.83
Corn gluten meal	1.93	2.50
Rapeseed meal	0.50	0.63
Fractionated vegetable oil ^2^	0.92	1.15
Soybean hulls	9.80	12.25
Wheat middlings	7.50	10.66
Rice bran	1.60	2.54
Molasses, beet sugar	2.00	2.38
Pellet binder ^3^	1.20	1.50
Lime stone	1.18	1.48
Premix-1 ^4^	0.94	1.18
Salt	0.45	0.56
Premix-2 ^5^	0.25	0.31
Live yeast ^6^	0.10	0.13
Nutrient compositions, % of dry matter		
Dry matter (% of as-fed)	88.08	88.3
Crude protein	22.25	25.81
Ether extract	4.26	4.59
Crude ash	8.30	10.13
Neutral detergent fiber	21.34	24.44
Acid detergent fiber	11.80	13.66
Acid detergent lignin	2.13	2.43

^1^ DDGS: corn dry distiller grain with soluble. ^2^ The fractionated vegetable oil (Evyap Oleo 1685, Evyap Sabun Malaysia Sdn Bhd Co., Johor, Malaysia) contained: minimum 85% of palmitic acid (C 16:0). ^3^ The pellet binder (Megabind 100, Tempe Chemical Feed Additives Co., İstanbul, Türkiye) contained (per kg): 999.40 mg of Bentonite, 200 mg of Guar gum, 200 mg of Xanthan gum, and 200 mg of Gum Arabic. ^4^ The premix (Premix Balance BSMX GRAN, Cargill Agriculture and Food Industry Co., Sakarya, Türkiye) contained (per kg): 430 g of calcium carbonate, 250 g of magnesium oxide, 200 g of sodium carbonate, and 120 g of Sea algae. ^5^ The premix (Kavimix VM, Kartal Chemical Co., Kocaeli, Türkiye) contained (per kg): 6,500,000 IU of vitamin A, 1,500,000 IU of vitamin D, 50,000 mg of vitamin E, 250,000 mg of choline, 9500 mg of niacin, 5000 mg of pantothenic acid, 3750 mg of riboflavin, 3750 mg of thiamin, 2500 mg of pyridoxine, 150 mg of folic acid, 50 mg of biotin, 40 mg of cyanocobalamin, 20,000 mg of Fe, 15,000 mg of Zn, 6000 mg of Mn, 2000 mg of Cu, 180 mg of I, 140 mg of Se, and 8 mg of Co. ^6^ The live yeast (Yea-Sacc Ts, Alltech Biotechnology Co., Dunboyne, Ireland) contained (per kg): 1 × 10^12^ cfu of *Saccharomyces cerevisiae* (CBS 493.94).

**Table 3:** Ingredient and chemical compositions of experimental starter diets.

Ingredient, % of as-Fed	PSS ^1^	PLS ^2^	PWCSS ^3^	PWCLS ^4^
Wheat straw	10	10	10	10
Pelleted starter	90	90	70	70
Whole corn	-	-	20	20
Chemical composition, % of dry matter				
Dry matter (% of as-fed)	88.41	88.41	88.39	88.39
Crude protein	20.33	20.33	20.42	20.42
Ether extract	4.01	4.01	4.19	4.19
Crude ash	8.31	8.31	8.43	8.43
Starch	32.38	32.38	31.01	31.01
Neutral detergent fiber	27.10	27.10	27.37	27.37
Acid detergent fiber	16.04	16.04	15.92	15.92
Acid detergent lignin	3.01	3.01	3.05	3.05
Non-fiber carbohydrate ^5^	40.36	40.36	40.61	40.61

^1^ PSS: total mixed ration containing 90% pelleted concentrate and 10% straw (short particle size). ^2^ PLS: total mixed ration containing 90% pelleted concentrate and 10% straw (long particle size). ^3^ PWCSS: total mixed ration containing 70% pelleted concentrate, 20% whole corn and 10% straw (short particle size). ^4^ PWCLS: total mixed ration containing 70% pelleted concentrate, 20% whole corn and 10% straw (long particle size). ^5^ Non-fiber-carbohydrate was calculated as [100 − (Neutral detergent fiber + Crude protein + ether extract + Crude ash)] [31].

**Table 4:** Effects of straw particle size (short or long) and physical forms of corn in starter diets on body weight, average daily weight gain, starter diet intake and feed efficiency of dairy calves (*n* = 15 calves per treatment).

	Treatments ^1^				SEM			
	SS		LS			*p*-Value ^2^		
Item	P	PWC	P	PWC		SPS	C	SPS × C
BW3 ^3^, kg	36.53	36.73	36.00	36.73	0.83	0.65	0.66	1.00
BW68 ^4^, kg	86.53 ^a^	90.39 ^a^	79.07 ^b^	86.28 ^a^	2.02	<0.01	<0.01	<0.01
ADG ^5^, kg/d	0.76 ^a^	0.81 ^a^	0.65 ^b^	0.75 ^a^	0.30	<0.01	<0.01	<0.01
FI ^6^, kg/d	0.49 ^ab^	0.52 ^a^	0.46 ^ab^	0.45 ^b^	0.02	<0.01	0.61	0.05
FE ^7^	0.65 ^ab^	0.64 ^ab^	0.71 ^a^	0.61 ^b^	0.03	0.50	<0.01	0.01

^1^ Starter diet containing 90% pelleted calf starter (P) and 10% straw with a short particle size (SS) on an as-fed basis (PSS); starter diet containing 70% pelleted calf starter plus 20% whole corn (PWC) and 10% straw with a short particle size on an as-fed basis (PWCSS); starter diet containing 90% pelleted calf starter (P) and 10% straw with a long particle size (LS) on an as-fed basis (PLS); starter diet containing 70% pelleted calf starter plus 20% whole corn (PWC) and 10% straw with a long particle size on an as-fed basis (PWCLS). ^2^ Statistical comparisons: SPS = straw particle size; C = physical form of corn in starter diets; SPS × C = interaction of straw particle size and physical form of corn in starter diets. ^3^ Body weight at 3 days of age. ^4^ Body weight at 68 days of age. ^5^ Average daily weight gain from 3 days to 68 days of age. ^6^ Mean starter feed (TMR) intake from 3 days to 68 days of age. ^7^ Feed efficiency = kg of mean starter feed (TMR) intake/kg of mean average daily weight gain. ^a,b^ Means within a row with different superscript letters are different (*p* ≤ 0.05).

**Table 5:** Effects of straw particle size (short or long) and physical forms of corn in starter diets on structural growth measurements (wither height and heart girth) of dairy calves (*n* = 15 calves per treatment).

	Treatments ^1^				SEM			
	SS		LS			*p*-Value ^2^		
Item	P	PWC	P	PWC		SPS	C	SPS × C
WH ^3^, cm								
d 3	78.33	78.13	78.27	78.20	0.53	1.00	0.92	1.00
d 68	93.13 ^a^	94.33 ^a^	89.20 ^b^	94.60 ^a^	1.04	0.02	<0.01	<0.01
Change	14.80 ^a^	16.20 ^a^	10.93 ^b^	16.40 ^a^	1.02	0.02	<0.01	<0.01
HG ^4^, cm								
d 3	79.27	78.80	79.20	79.00	0.32	0.77	0.29	0.92
d 68	102.73 ^b^	107.53 ^a^	102.07 ^b^	105.27 ^a^	1.14	0.08	<0.01	<0.01
Change	23.47 ^b^	28.73 ^a^	22.87 ^b^	26.27 ^a^	1.15	0.07	<0.01	<0.01

^1^ Starter diet containing 90% pelleted calf starter (P) and 10% straw with a short particle size (SS) on an as-fed basis (PSS); starter diet containing 70% pelleted calf starter plus 20% whole corn (PWC) and 10% straw with a short particle size on an as-fed basis (PWCSS); starter diet containing 90% pelleted calf starter (P) and 10% straw with a long particle size (LS) on an as-fed basis (PLS); starter diet containing 70% pelleted calf starter plus 20% whole corn (PWC) and 10% straw with a long particle size on an as-fed basis (PWCLS). ^2^ Statistical comparisons: SPS = straw particle size; C = physical form of corn in starter diets; SPS × C = interaction of straw particle size and physical form of corn in starter diets. ^3^ Wither heights at 3 days of age and 68 days of age. ^4^ Heart girths at 3 days of age and 68 days of age. ^a,b^ Means within a row with different superscript letters are different (*p* ≤ 0.05).

**Table 6:** Effects of straw particle size (short or long) and physical forms of corn in starter diets on ruminal pH and volatile fatty acid (VFA) concentrations of dairy calves (*n* = 15 calves per treatment).

	Treatments ^1^				SEM			
	SS		LS			*p*-Value ^2^		
Item	P	PWC	P	PWC		SPS	C	SPS × C
pH ^3^	6.06	5.80	5.76	5.81	0.13	0.12	0.13	0.15
A ^4^								
mmol/L	33.16	36.72	35.91	33.23	2.12	0.80	0.12	0.58
% of total VFA	50.27	47.06	49.82	52.05				
P ^5^								
mmol/L	23.94 ^bc^	29.41 ^a^	26.12 ^ab^	21.01 ^c^	1.81	0.02	<0.01	<0.01
% of total VFA	36.27	37.45	36.42	32.93				
B ^6^								
mmol/L	8.84 ^b^	12.12 ^a^	9.64 ^b^	9.53 ^b^	0.65	0.06	<0.01	<0.01
% of total VFA	13.46	15.50	13.76	15.02				
T ^7^, mmol/L	65.94 ^b^	78.25 ^a^	71.67 ^ab^	63.77 ^b^	3.61	0.09	<0.01	<0.01

^1^ Starter diet containing 90% pelleted calf starter (P) and 10% straw with a short particle size (SS) on an as-fed basis (PSS); starter diet containing 70% pelleted calf starter plus 20% whole corn (PWC) and 10% straw with a short particle size on an as-fed basis (PWCSS); starter diet containing 90% pelleted calf starter (P) and 10% straw with a long particle size (LS) on an as-fed basis (PLS); starter diet containing 70% pelleted calf starter plus 20% whole corn (PWC) and 10% straw with a long particle size on an as-fed basis (PWCLS). ^2^ Statistical comparisons: SPS = straw particle size; C = physical form of corn in starter diets; SPS × C = interaction of straw particle size and physical form of corn in starter diets. ^3^ pH values at 68 days of age. ^4^ Ruminal acetate concentrations at 68 days of age. ^5^ Ruminal propionate concentrations at 68 days of age. ^6^ Ruminal butyrate concentrations at 68 days of age. ^7^ Total VFA (acetate + propionate + butyrate) concentrations at 68 days of age. ^a,b,c^ Means within a row with different superscript letters are different (*p* ≤ 0.05).

**Table 7:** Effects of straw particle size (short or long) and physical forms of corn in starter diets on blood β-hydroxy butyrate (BHB) concentration and fecal score of dairy calves (*n* = 15 calves per treatment).

	Treatments ^1^				SEM			
	SS		LS			*p*-Value ^2^		
Item	P	PWC	P	PWC		SPS	C	SPS × C
BHB ^3^, mmol/L	0.16	0.16	0.17	0.13	0.02	0.66	0.18	0.41
Fecal score ^4^	1.41 ^c^	1.82 ^ab^	1.69 ^b^	2.07 ^a^	0.10	<0.01	<0.01	<0.01

^1^ Starter diet containing 90% pelleted calf starter (P) and 10% straw with a short particle size (SS) on an as-fed basis (PSS); starter diet containing 70% pelleted calf starter plus 20% whole corn (PWC) and 10% straw with a short particle size on an as-fed basis (PWCSS); starter diet containing 90% pelleted calf starter (P) and 10% straw with a long particle size (LS) on an as-fed basis (PLS); starter diet containing 70% pelleted calf starter plus 20% whole corn (PWC) and 10% straw with a long particle size on an as-fed basis (PWCLS). ^2^ Statistical comparisons: SPS = straw particle size; C = physical form of corn in starter diets; SPS × C = interaction of straw particle size and physical form of corn in starter diets. ^3^ The concentrations of blood β-hydroxy butyrate at 68 days of age. ^4^ Fecal samples were scored daily with regard to consistency according to the following system: 1 = normal, 2 = soft to loose, 3 = loose to watery, 4 = watery, mucous, slightly bloody, and 5 = watery, mucous and bloody [33]. ^a,b,c^ Means within a row with different superscript letters are different (*p* ≤ 0.05).

## Data Availability

The original contributions presented in this study are included in the article. Further inquiries can be directed to the corresponding author.
